# Coexistence of Anaemia and Common Morbidities Among Children in India Below the Age of Five Years: Evidence From the National Family Health Survey-5 (2019-21)

**DOI:** 10.7759/cureus.109057

**Published:** 2026-05-17

**Authors:** Saurabh Kashyap, Abhishek Singh, Monika Agarwal, Akshata M. A., Prashant Singh

**Affiliations:** 1 Community Medicine and Public Health, King George's Medical University, Lucknow, IND; 2 Quality Assurance, Uttar Pradesh National Health Mission, Lucknow, IND

**Keywords:** acute respiratory infection, childhood anaemia, childhood morbidities, diarrhoea, fever

## Abstract

Introduction

Childhood anaemia remains one of the most persistent public health problems in India. At the same time, common childhood morbidities such as diarrhoea, fever, and acute respiratory infection continue to affect a large proportion of children under five. This study, therefore, aims to examine the association between anaemia and common morbidities among children in India.

Material and methods

This cross-sectional study used the fifth nationally representative National Family Health Survey (NFHS-5) data for children aged 6-59 months born in the five years preceding the survey conducted in India during 2019-21. The analysis included 179,262 children. Three common morbidities were considered: acute respiratory infection (ARI), fever, and diarrhoea reported in the two weeks preceding the survey. Anaemia status was assessed using haemoglobin measurement. Bivariate associations between anaemia and covariates were examined using chi-square tests. Binary logistic regression models were fitted in four stages to estimate the adjusted association between child morbidities and anaemia.

Results

The overall prevalence of anaemia among children aged 6-59 months was high, with about two-thirds of children classified as anaemic. The prevalence of recent morbidity was 2.8% for ARI, 13.7% for fever, and 7.4% for diarrhoea. In bivariate analysis, children with ARI (69.9%), fever (70.1%), and diarrhoea (73.9%) had significantly higher anaemia prevalence than those without these conditions. In multivariable analysis, diarrhoea remained independently associated with anaemia after full adjustment (OR: 1.09, 95% CI: 1.04-1.14, p < 0.001), while fever showed a smaller but significant association (OR: 1.06, 95% CI: 1.02-1.10, p = 0.001). The attenuation of ARI (OR: 1.03, 95% CI: 0.97-1.10, p = 0.354) after adjustment suggests that the observed crude association may largely reflect shared socio-economic and nutritional vulnerabilities rather than an independent relationship. Children aged 24-41 months and 42-59 months have substantially lower odds of anaemia (OR: 0.57, 95% CI: 0.56-0.59, p < 0.001) and (OR: 0.33, 95% CI: 0.32-0.34, p < 0.001), respectively, than those aged 6-23 months. Children born to mothers less than 18 years were at higher risk of anaemia (OR: 1.30, 95% CI: 1.19-1.41, p < 0.001). The likelihood of being anaemic was highest among children born to mothers with no schooling (OR: 1.44, 95% CI: 1.39-1.50, p < 0.001) compared with children born to mothers with 12 or more years of schooling. Higher odds of any anaemia were observed among children from the poorest households (OR: 1.17, 95% CI: 1.11-1.23, p < 0.001) and in several high-burden regions. Predicted probability estimates showed that the probability of anaemia was higher among children with ARI (70.5%), fever (69.7%), and especially diarrhoea (73.0%) compared with children without these morbidities.

Conclusion

The study demonstrates that anaemia among Indian children is not only highly prevalent but also closely linked with common childhood morbidities, particularly diarrhoea. Special attention is needed for children in the first two years of life, when vulnerability is highest. These findings suggest that anaemia control strategies in India should be integrated with stronger prevention and treatment of childhood infections, improved maternal education, and targeted support for the poorest and most disadvantaged in high-burden regions.

## Introduction

Childhood anaemia and common childhood morbidities continue to be significant public health challenges in low- and middle-income countries (LMICs). About one-third of children aged 6-59 months worldwide are anaemic, with the highest prevalence found in sub-Saharan Africa and Southeast Asia [1,2]. Childhood anaemia is associated with impeded cognitive and motor development, as well as increased susceptibility to illness. Despite global commitments to reduce childhood anaemia, recent studies indicate that progress has been slow and inconsistent [3]. India significantly contributes to the global prevalence of childhood anaemia. The fifth National Family Health Survey (NFHS-5), 2019-21, shows that about two-thirds of children aged 6-59 months are anaemic [4,5]. The national prevalence estimates are around 67%, an increase of about 9 percentage points since the fourth National Family Health Survey (NFHS-4) carried out in 2015-16 [6]. Many studies have shown significant variability across states, with higher prevalence found in rural regions and among socio-economically disadvantaged populations [7]. Recent geospatial analyses at the district level utilizing NFHS-5 data corroborate significant spatial clustering [8,9]. A recent systematic review and meta-analysis demonstrates a consistently elevated prevalence (60-70%) of anaemia among children in India [9].

Common acute morbidities, especially acute respiratory infections (ARI), fever, and diarrhoea, continue to impose a substantial burden on children in India. Despite the National Family Health Survey (NFHS) rounds having shown improvements in under-five mortality rates, diarrhoeal diseases and ARI are still the main causes of illness and death [5,10,11]. There exist significant biological and epidemiological connections between anaemia and infection. Childhood anaemia can compromise both cell-mediated and humoral immunity, increasing vulnerability to respiratory and gastrointestinal diseases. Conversely, recurrent infections can exacerbate anaemia or initiate it by reducing appetite, impairing iron absorption, or causing blood loss [8,12].

Many studies in India have found that children who have recently had diarrhoea or a fever are more likely to be anaemic. This suggests a strong link between acute infections and haemoglobin levels [13-15]. A recent NFHS-based study on Indian children aged 6-59 months found that recent diarrhoea and fever are significant predictors of moderate-to-severe anaemia [16]. Research from LMICs has identified significant comorbidity between anaemia and stunting, suggesting syndemic frameworks [17]. Recent studies in South Asia, including Nepal, have measured the coexistence of undernutrition and anaemia in children aged 6-59 months [18]. However, although these studies clarify the coexistence of chronic undernutrition and anaemia, evidence on the coexistence of anaemia with acute morbidities, such as diarrhoea and ARI in large, nationally representative samples remains limited [4,7,8,10,14]. There is still not enough national-level evidence showing the number of children under five years of age with both anaemia and common childhood diseases, or how this varies by bio-demographic and socio-economic variables. Addressing this gap is essential for enhancing child health and nutrition strategies, as children concurrently affected by anaemia and acute infections may face heightened mortality risk. Therefore, this study uses NFHS-5 (2019-21) [19] data to investigate the coexistence of anaemia and prevalent morbidities, specifically diarrhoea, fever, and ARI symptoms among children aged 6-59 months in India. The objective is to determine the association between childhood anaemia and childhood morbidities, while adjusting for bio-demographic and socio-economic variables.

## Materials and methods

Data and study population

This study uses publicly accessible, de-identified data from NFHS-5, conducted in India during 2019-21 [19]. The survey is nationally representative, providing information at both district and state levels. Villages in rural regions and census enumeration wards in urban regions served as primary sampling units (PSUs). Of 259,627 births in the five years before the survey, 179,262 children were aged 6-59 months. Only these children were included in the analysis because data on childhood anaemia were available only for this age group. The information on haemoglobin levels, ARI, fever, and childhood diarrhoea was available for all 179,262 children. Therefore, 179,262 children aged 6-59 months had all the necessary information for the study and were included in the analysis.

Outcome variable

In NFHS-5, the prevalence of anaemia was estimated using haemoglobin levels and adjusted for altitude by the Centers for Disease Control and Prevention (CDC) formulas [20]. Haemoglobin levels are reported in grams per deciliter (g/dl). In this study, haemoglobin levels were grouped into two categories: haemoglobin ≤11.0 g/dl (anaemia status: yes) and haemoglobin >11.0 g/dl (anaemia status: no) [19].

Explanatory variables

Childhood Morbidities Variables

Child morbidity characteristics were the primary explanatory variables. In NFHS-5, mothers of children born in the five years before the survey were asked if their children had cough, fever, or diarrhoea in the two weeks before the survey. The accuracy depends on the mothers’ recall of the illness's onset. A two-week recall period was adopted to ensure enough cases and reduce recall errors. Childhood morbidity variables included ARI, fever, and diarrhoea in the last two weeks (no/yes). ARI symptoms include cough with short, rapid, or difficult chest-related breathing [19].

Bio-Demographic Variables

The bio-demographic characteristics in this study included: age of the child in months (6-23, 24-41, 42-59), sex of the child (female, male), and birth order (first, second, three and more). Preterm birth (no, yes) and size of child at birth (average, larger than average, smaller than average, very large, very small) were also included. At the mother level, the following were included: mother’s age at delivery (less than 18 years, 18-24 years, 25-34 years, 35-49 years), mother’s education (12 or more years complete, 10-11 years complete, 8-9 years complete, 5-7 years complete, less than 5 years complete, and no schooling), and regular exposure to any mass media (yes, no).

Socio-Economic Variables

Socio-economic factors analyzed were: residence (urban/rural), religion (Hindu, Muslim, others), caste (none, other backward castes (OBCs), scheduled tribes (STs), scheduled castes (SCs), don’t know), and wealth index (richest, richer, middle, poorer, poorest). NFHS household wealth quintiles are based on ownership and housing conditions. National data is subdivided into six regions: south, west, northeast, east, central, and north. The south has five states and three union territories (Andaman & Nicobar Islands, Andhra Pradesh, Karnataka, Kerala, Lakshadweep, Puducherry, Tamil Nadu, and Telangana). The west consists of three states and one union territory (Dadra & Nagar Haveli and Daman & Diu, Goa, Gujarat, and Maharashtra). The northeast comprises eight states (Arunachal Pradesh, Assam, Manipur, Meghalaya, Mizoram, Nagaland, Sikkim, and Tripura). The east region includes four states (Bihar, Jharkhand, Odisha, and West Bengal). The central region has three states (Chhattisgarh, Madhya Pradesh, and Uttar Pradesh). The northern region consists of five states and four union territories (Chandigarh, Delhi, Haryana, Himachal Pradesh, Jammu & Kashmir, Ladakh, Punjab, Rajasthan, and Uttarakhand).

Statistical analysis

The study used univariate, bivariate, and multivariate analyses. Univariate analysis calculated the percentage distribution of respondents by background characteristics. Chi-square tests were used to analyse the bivariate relationships between explanatory variables and anaemia categories among children. The outcome variable was anaemia status among children aged 6-59 months (no/yes). Since the outcome variable is dichotomous, bivariate and multivariate binary logistic regression models were used. These examined the effect of childhood morbidity variables on the outcome. Regression outcomes were shown as unadjusted odds ratios (UOR) and adjusted odds ratios (AOR), with 95% confidence intervals (CI) and p-values. Predicted probabilities [21] were also computed for child-morbidity variables to assess their influence. The statistical analysis accounted for survey weights, clustering, and stratification in the sample design. All statistical analyses were done in R version 4.5.1 (R Foundation for Statistical Computing, Vienna, Austria) [22]. A p-value of <0.05 was considered significant.

Ethics

The statistical analysis was conducted anonymously, utilizing publicly available secondary data and without any identifiable information on the survey participants, for which ethical approval is not required. NFHS-5 data has been used in accordance with the Demographic and Health Survey (DHS) guidelines, ensuring data confidentiality, secure storage, and no attempts to identify individuals, households, or communities.

## Results

Distribution by childhood morbidity, bio-demographic, and socio-economic variables

Table 1 describes the percentage distribution of children aged 6-59 months by childhood morbidity, bio-demographic, and socio-economic characteristics in India. Only about 2.8% of children were reported to have symptoms of ARI, 7.4% had diarrhoea, and 13.7% had fever in the two weeks preceding the survey. The children were evenly distributed across the three age categories, with one‑third in each of the 6-23, 24-41, and 42-59 month age groups. There was a slight predominance of male children (51.9%) compared with females (48.1%). The first-order births accounted for the largest share (38.7%), followed by second-order births (34.1%), while about 27.2% of children were in third- or higher-order births. Most children were reported as not being preterm (87.8%), and a large majority were perceived by their mothers as having average birth size (70.6%). 

**Table 1:** Distribution of study population by childhood morbidity, bio-demographic (child and mother), and socio-economic variables in India, NFHS-5 (2019–2021) NFHS: National Family Health Survey

Variables		Frequency	Percentage	95% CI
Childhood Morbidity Variables				
Acute respiratory infection (ARI)	No	174,165	97.2	97.1-97.2
	Yes	5,097	2.84	2.77-2.92
Fever	No	154,639	86.3	86.1-86.4
	Yes	24,623	13.7	13.6-13.9
Diarrhoea	No	166,068	92.6	92.5-92.8
	Yes	13,194	7.36	7.24-7.48
Bio-Demographic Variables				
Child level variables				
Age of the child (in months)	6-23	58,241	32.5	32.3-32.7
	24-41	60,397	33.7	33.5-33.9
	42-59	60,624	33.8	33.6-34.0
Sex of the child	Female	86,203	48.1	47.9-48.3
	Male	93,059	51.9	51.7-52.1
Birth order	Second	61,135	34.1	33.9-34.3
	First	69,308	38.7	38.4-38.9
	Third or higher	48,819	27.2	27.0-27.4
Preterm birth	No	157,444	87.8	87.7-88.0
	Yes	21,818	12.2	12.0-12.3
Size of child at birth	Average	125,362	70.6	70.4-70.8
	Larger than average	21,014	11.8	11.7-12.0
	Smaller than average	14,202	8.00	7.88-8.13
	Very Large	12,698	7.15	7.03-7.28
	Very Small	4,212	2.37	2.30-2.44
Mother level variables				
Mother's age at delivery (in years)	35-49	6,059	3.38	3.30-3.46
	25-34	70,787	39.5	39.3-39.7
	18-24	97,116	54.2	53.9-54.4
	<18	5,300	2.96	2.88-3.04
Mother's education	12 or more years complete	48,075	26.8	26.6-27.0
	10-11 years complete	24,084	13.4	13.3-13.6
	8-9 years complete	33,369	18.6	18.4-18.8
	5-7 years complete	26,257	14.6	14.5-14.8
	<5 years complete	8,838	4.93	4.83-5.03
	No schooling	38,640	21.6	21.4-21.7
Mother regularly exposed to any mass media	Yes	93,748	52.3	52.1-52.5
	No	85,514	47.7	47.5-47.9
Socio-Economic Variables				
Place of residence	Urban	47,031	26.2	26.0-26.4
	Rural	132,231	73.8	73.6-74.0
Religion	Hindu	143,019	79.8	79.6-80.0
	Muslim	28,411	15.8	15.7-16.0
	Others	7,831	4.37	4.27-4.46
Caste	None of them	31,700	18.5	18.4-18.7
	Other Backward Castes	77,612	45.4	45.2-45.6
	Schedule Tribes	18,180	10.6	10.5-10.8
	Schedule Castes	41,862	24.5	24.3-24.7
	Don't know	1,583	0.926	0.882-0.973
Wealth Index	Richest	27,503	15.3	15.2-15.5
	Richer	33,121	18.5	18.3-18.7
	Middle	35,395	19.7	19.6-19.9
	Poorer	39,183	21.9	21.7-22.1
	Poorest	44,060	24.6	24.4-24.8
Region	South	29,871	16.7	16.5-16.8
	West	22,948	12.8	12.6-13.0
	Northeast	6,895	3.85	3.76-3.94
	East	48,069	26.8	26.6-27.0
	Central	47,609	26.6	26.4-26.8
	North	23,870	13.3	13.2-13.5
Total		179,262	-	-

Maternal characteristics show that more than half of the births occurred to mothers aged 18-24 years at the time of delivery (54.2%). About 26.8% of mothers had completed 12 or more years of schooling, and only 21.6% had never attended school. Just over half of mothers (52.3%) reported regular exposure to at least one form of mass media. Among socio-economic characteristics, nearly three‑quarters of the children resided in rural areas (73.8%), and the majority belonged to Hindu households (79.8%), followed by Muslim (15.8%). Children from OBC formed the largest group (45.4%), followed by those from SCs (24.5%) and those reporting none of them (18.5%). The wealth index distribution shows that children were more concentrated in the poorer and poorest quintiles (21.9% and 24.6%, respectively) than in the richer and richest quintiles (18.5% and 15.3%, respectively). The eastern and central regions together accounted for more than half of the births (26.8% and 26.6%, respectively), followed by the south (16.7%), the north (13.3%), the west (12.8%), and a smaller share from the northeast (3.9%). 

Association of childhood morbidity, bio-demographic, and socio-economic variables with anaemia status

Table 2 presents the associations between anaemia status and child morbidity, bio-demographic, and socio-economic characteristics among children aged 6-59 months in India. Overall, about two‑thirds of the children in the study were anaemic (68.0%). The proportion of anaemia was higher among children with ARI (69.9%; chi-square statistic: 21.9; p<0.001), those with fever in the last two weeks (70.1%; chi-square statistic: 78.8; p<0.001), and especially those with diarrhoea (73.9%; chi-square statistic: 209.0; p<0.001) compared to their counterparts without these conditions. The prevalence of anaemia was highest among children aged 6-23 months (79.0%), followed by those aged 24-41 months (68.9%) and those aged 42-59 months (56.6%), with a chi-square statistic value of 6992.0 and p-value of p<0.001. The prevalence of anaemia was identical among boys and girls (68.0% each; chi-square statistic: 0.839; p=0.360). The higher birth order was associated with a greater anaemia burden, increasing from 66.2% among first‑order births to 67.9% in second‑order births and 70.7% among third-order and higher births (chi-square statistic: 101.0; p<0.001). Children born preterm had slightly higher anaemia prevalence (69.0%) than those not reported as preterm (67.9%; chi-square statistic: 8.81; p=0.003). Perceived size at birth showed a graded association, with anaemia prevalence rising from 67.7% among children of average size to 70.8% among those reported as very small at birth (chi-square statistic: 58.2; p<0.001). 

**Table 2:** Association of anaemia status in study population with childhood morbidity, bio-demographic, and socio-economic variables in India, NFHS-5 (2019–2021) NFHS: National Family Health Survey

Variables		Anaemia Status		Chi-square statistic	p-value
		No	Yes		
Childhood Morbidity Variables					
Acute respiratory infection (ARI)	No	32.0	68.0	21.9	<0.001
	Yes	30.1	69.9		
Fever	No	32.3	67.7	78.8	<0.001
	Yes	29.9	70.1		
Diarrhoea	No	32.4	67.6	209.0	<0.001
	Yes	26.1	73.9		
Bio-Demographic Variables					
Child level variables					
Age of the child (in months)	6-23	21.0	79.0	6992.0	<0.001
	24-41	31.1	68.9		
	42-59	43.4	56.6		
Sex of the child	Female	32.0	68.0	0.839	0.360
	Male	32.0	68.0		
Birth order	Second	32.1	67.9	101.0	<0.001
	First	33.8	66.2		
	Third or higher	29.3	70.7		
Preterm birth	No	32.1	67.9	8.81	0.003
	Yes	31.0	69.0		
Size of child at birth	Average	32.3	67.7	58.2	<0.001
	Larger than average	31.8	68.2		
	Smaller than average	31.2	68.8		
	Very large	31.1	68.9		
	Very small	29.2	70.8		
Mother level variables					
Mother's age at delivery (in years)	35-49	34.5	65.5	96.6	<0.001
	25-34	32.3	67.7		
	18-24	31.7	68.3		
	<18	30.2	69.8		
Mother's education	12 or more years complete	37.2	62.8	931.0	<0.001
	10-11 years complete	33.6	66.4		
	8-9 years complete	31.2	68.8		
	5-7 years complete	29.5	70.5		
	<5 years complete	29.5	70.5		
	No schooling	27.4	72.6		
Mother regularly exposed to any mass media	Yes	34.4	65.6	385.0	<0.001
	No	29.3	70.7		
Socio-Economic Variables					
Place of residence	Urban	35.2	64.8	192.0	<0.001
	Rural	30.8	69.2		
Religion	Hindu	31.6	68.4	1829.0	<0.001
	Muslim	32.3	67.7		
	Others	37.2	62.8		
Caste	None of them	34.2	65.8	327.0	<0.001
	Other Backward Castes	33.8	66.2		
	Schedule Tribes	26.1	73.9		
	Schedule Castes	29.6	70.4		
	Don't know	25.9	74.1		
Wealth Index	Richest	37.2	62.8	516.0	<0.001
	Richer	35.2	64.8		
	Middle	32.4	67.6		
	Poorer	30.2	69.8		
	Poorest	27.5	72.5		
Region	South	39.5	60.5	2805.0	<0.001
	West	26.7	73.3		
	Northeast	35.8	64.2		
	East	30.7	69.3		
	Central	31.5	68.5		
	North	30.0	70.0		
Total		57,311	121,951	-	-

Among maternal characteristics, the prevalence of anaemia was marginally higher among children of younger mothers (<18 years: 69.8%; 18-24 years: 68.3%) compared with those born to mothers aged 35-49 years (65.5%; chi-square statistic: 96.6; p<0.001). There was a pronounced inverse relationship with maternal education; prevalence of anaemia declined from 72.6% among children of mothers with no schooling to 70.5-70.5% in those with less than primary or primary schooling, and further to 62.8% among children of mothers with 12 or more years of education (chi-square statistic: 931.0; p<0.001). Children of mothers who were regularly exposed to any mass media had a lower prevalence of anaemia (65.6%) than those whose mothers were not exposed to any mass media (70.7%), with a chi-square statistic value of 385.0 and p-value of <0.001. 

The socio-economic and contextual variables also showed strong associations with anaemia status among children aged 6-59 months. Rural children had a higher prevalence of anaemia (69.2%) than urban children (64.8%; chi-square statistic: 192.0; p<0.001). By religion, anaemia was most common among Hindu children (68.4%), slightly lower among Muslims (67.7%), and lowest among children from other religions (62.8%; chi-square statistic: 1829.0; p<0.001). The significant caste differences were observed, with a higher prevalence of anaemia among children from ST (73.9%) and SC (70.4%) compared with those from OBC (66.2%) and children not belonging to these groups (65.8%; chi-square statistic: 327.0; p<0.001). A clear wealth gradient emerged, with anaemia prevalence decreasing steadily from 72.5% in the poorest quintile to 62.8% in the richest quintile (chi-square statistic: 516.0; p<0.001). Regional patterns indicated the lowest prevalence in the southern region (60.5%) and comparatively higher levels in the west (73.3%), north (70.0%), east (69.3%), central (68.5%), and to a lesser extent the northeast (64.2%; chi-square statistic: 2805.0; p<0.001).

Bivariate and multivariate binary logistic regression analysis of anaemia status

ORs and aORs from a series of binary logistic regression models examining the association between childhood morbidity, bio-demographic, and socio-economic factors and anaemia status in the study population are presented as Models 1-4. Model 1 includes only morbidity variables, Model 2 additionally adjusts for child‑level characteristics, and Model 3 further adjusts for maternal factors (for Models 1-3, see Appendices). Model 4 is the fully adjusted model that is a combination of Model 3 with the inclusion of socio-economic variables (Table 3). Regarding morbidity, all three conditions show positive associations with anaemia status in the unadjusted model, but their strength and significance change with adjustment. For ARI, the odds of anaemia are higher in Model 1 (OR 1.17; 95% CI 1.10-1.25), but this association becomes non‑significant after adjusting for child, maternal, and socio-economic factors (Model 4: OR 1.03; 95% CI 0.97-1.10; p=0.354). Fever remains associated with anaemia status in the fully adjusted model (Model 4: OR 1.06; 95% CI 1.02-1.10; p=0.001), indicating a small but statistically significant increase in odds. Diarrhoea shows the most consistent and robust association; children with diarrhoea have about 9% higher odds of anaemia than those without diarrhoea (Model: 4 OR 1.09; 95% CI 1.04-1.14; p<0.001).

**Table 3:** Fully adjusted binary logistic regression estimates of the effect of childhood morbidity variables on the prevalence of anaemia in the study population by adjusting for the bio-demographic and socio-economic factors (Model 4) Note: Models 1-3 can be seen in the Appendices; Model 4 = Model 3 + socio-economic factors ref.: reference category

Variables		OR	95% CI	p-value
Childhood morbidity variables				
Acute respiratory infection (ARI)	No (ref.)	1.00	-	-
	Yes	1.03	0.97-1.10	0.354
Fever	No (ref.)	1.00	-	-
	Yes	1.06	1.02-1.10	0.001
Diarrhoea	No (ref.)	1.00	-	-
	Yes	1.09	1.04-1.14	<0.001
Bio-Demographic Variables				
Child-level variables				
Age of the child (in months)	6-23 (ref.)	1.00	-	-
	24-41	0.57	0.56-0.59	<0.001
	42-59	0.33	0.32-0.34	<0.001
Sex of the child	Female (ref.)	1.00		
	Male	1.01	0.99-1.03	0.463
Birth order	Second (ref.)	1.00		
	First	0.96	0.94-0.98	0.002
	Third or higher	1.02	0.99-1.05	0.124
Preterm birth	No (ref.)	1.00		
	Yes	1.01	0.98-1.05	0.477
Size of child at birth	Average (ref.)	1.00		
	Larger than average	1.05	1.02-1.09	0.002
	Smaller than average	1.06	1.01-1.10	0.008
	Very large	1.05	1.00-1.09	0.036
	Very small	1.12	1.04-1.20	0.004
Mother level variables				
Mother's age at delivery (in years)	35-49 (ref.)	1.00	-	-
	25-34	1.13	1.07-1.19	<0.001
	18-24	1.21	1.14-1.28	<0.001
	<18	1.30	1.19-1.41	<0.001
Mother's education	12 or more years complete (ref.)	1.00	-	-
	10-11 years complete	1.12	1.08-1.16	<0.001
	8-9 years complete	1.17	1.13-1.21	<0.001
	5-7 years complete	1.25	1.21-1.30	<0.001
	<5 years complete	1.30	1.24-1.38	<0.001
	No schooling	1.44	1.39-1.50	<0.001
Mother regularly exposed to any mass media	Yes (ref.)	1.00	-	-
	No	1.04	1.02-1.07	<0.001
Socio-Economic Variables				
Place of residence	Urban (ref.)	1.00	-	-
	Rural	1.04	1.01-1.07	0.005
Religion	Hindu (ref.)	1.00	-	-
	Muslim	1.02	0.99-1.06	0.266
	Others	0.65	0.62-0.67	<0.001
Caste	None of them (ref.)	1.00	-	-
	OBCs	1.03	1.00-1.06	0.055
	Schedule Tribes	1.28	1.23-1.33	<0.001
	Schedule Castes	1.20	1.16-1.24	<0.001
	Don't know	1.30	1.13-1.49	<0.001
Wealth Index	Richest (ref.)	1.00	-	-
	Richer	1.03	0.99-1.07	0.143
	Middle	1.09	1.04-1.13	<0.001
	Poorer	1.09	1.04-1.14	<0.001
	Poorest	1.17	1.11-1.23	<0.001
Region	South (ref.)	1.00	-	-
	West	1.87	1.78-1.96	<0.001
	Northeast	0.78	0.74-0.81	<0.001
	East	1.17	1.13-1.22	<0.001
	Central	1.24	1.19-1.28	<0.001
	North	1.43	1.38-1.49	<0.001

Among child-level bio-demographic variables, there is a strong inverse gradient with age. Compared with children aged 6-23 months, those aged 24-41 months have significantly lower odds of anaemia (Model 4: OR 0.57; 95% CI 0.56-0.59), and those aged 42-59 months have even lower odds (OR 0.33; 95% CI 0.32-0.34; both p<0.001). Sex of the child is not associated with anaemia status in any model (Model 4: OR for males 1.01; 95% CI 0.99-1.03; p=0.463). Compared with second‑order births, first‑order children have slightly lower odds of anaemia (Model 4: OR 0.96; 95% CI 0.94-0.98). Children of birth order three or more have similar or marginally higher odds in partially adjusted models, but the association weakens and becomes nonsignificant in the fully adjusted model (Model 4: OR 1.02; 95% CI 0.99-1.05; p=0.124). Preterm birth is associated with a slightly increased risk of anaemia in Model 2 (OR 1.04; 95% CI 1.00-1.07; p=0.026), but this effect attenuates and becomes non‑significant after further adjustment (Model 4: OR 1.01; 95% CI 0.98-1.05; p=0.477). Perceived size at birth, however, remains an independent predictor; compared with children of average size, those reported as smaller than average (OR 1.06; 95% CI 1.01-1.10), very small (OR 1.12; 95% CI 1.04-1.20), larger than average (OR 1.05; 95% CI 1.02-1.09) and very large (OR 1.05; 95% CI 1.00-1.09) all have significantly higher odds of anaemia in Model 4 (all p<0.05).

Among mother-level variables, compared with mothers aged 35-49 years at delivery, children of adolescent mothers (<18 years) have 30% higher odds of anaemia (Model 4: OR 1.30; 95% CI 1.19-1.41), and those of mothers aged 18-24 years and 25-34 years also have higher odds (OR 1.21 and 1.13 respectively; all p<0.001), even after full adjustment. Maternal education demonstrates a clear inverse gradient; relative to mothers with 12 years of schooling, the odds of anaemia rise progressively across education categories, from OR 1.12 (10-11 years) and 1.17 (8-9 years) to 1.25 (5-7 years), 1.30 (5 years), and highest OR 1.44 (95% CI 1.39-1.50) for mothers with no schooling in Model 4 (all p<0.001). Children of mothers not regularly exposed to any mass media also have higher odds of anaemia than those whose mothers are exposed (Model 4: OR 1.04; 95% CI 1.02-1.07; p<0.001), underscoring the role of information and awareness.

The socio-economic variables retain significant associations even after extensive adjustment for morbidity, and child and maternal characteristics. Rural residence is associated with slightly higher odds of anaemia than urban residence (Model 4: OR 1.04; 95% CI 1.01-1.07; p=0.005). By religion, Muslim children do not differ significantly from Hindu children (OR 1.02; 95% CI 0.99-1.06). On the other hand, children from other religions have substantially lower odds (OR 0.65; 95% CI 0.62-0.67; p<0.001). Compared with children from the "none of them" (reference) category of caste, those from ST (OR 1.28; 95% CI 1.23-1.33) and SC (OR 1.20; 95% CI 1.16-1.24) have significantly higher odds of anaemia. On the other hand, association with OBCs is marginal (OR 1.03; 95% CI 1.00-1.06; p=0.055); children with unknown caste also show elevated risk (OR 1.30; 95% CI 1.13-1.49; p<0.001). Relative to the richest quintile, odds of anaemia increase progressively across the middle (OR 1.09; 95% CI 1.04-1.13), poorer (OR 1.09; 95% CI 1.04-1.14), and poorest (OR 1.17; 95% CI 1.11-1.23) wealth groups in Model 4 (all p<0.001). On the other hand, the richer group is not significantly different (OR 1.03; 95% CI 0.99-1.07). Using the southern region as reference, children in the west (OR 1.87; 95% CI 1.78-1.96), north (OR 1.43; 95% CI 1.38-1.49), central (OR 1.24; 95% CI 1.19-1.28) and east (OR 1.17; 95% CI 1.13-1.22) have substantially higher odds of anaemia, whereas those in the northeast actually have lower odds (OR 0.78; 95% CI 0.74-0.81; all p<0.001).

Predicted probabilities for anaemia status by child morbidity variables

**Figure 1:**
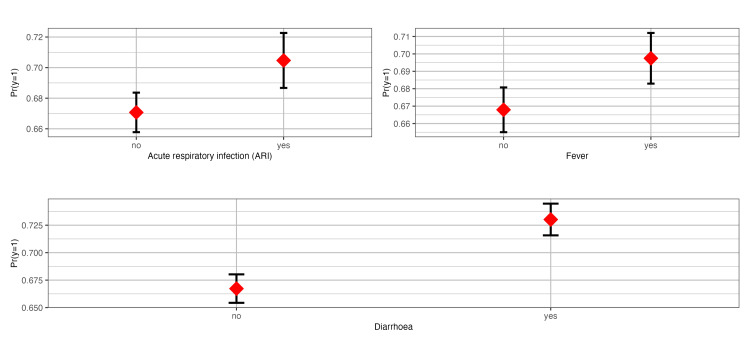
Predicted probabilities for anaemia status among children by childhood morbidity variables

Figure 1 presents the model-based (Model 4) predicted probabilities of anaemia status among children, stratified by the presence of recent acute morbidities. For all three conditions, ARI, fever, and diarrhoea, the predicted probability of anaemia is higher among children who experienced the morbidity compared to those who did not. The predicted probability of anaemia among children with ARI is 70.5% (95% CI: 68.7-72.3) versus 67.1% (65.8-68.4) among those without ARI, indicating about a 3-4 percentage‑point excess risk associated with respiratory symptoms. Similarly, the predicted probability of anaemia among children with fever is 69.7% (68.3-71.2), compared with 66.8% (65.5-68.1) among those without fever, again suggesting a modest but meaningful elevation in risk. Children with diarrhoeal episodes have an estimated 73.0% (71.6-74.5) probability of being anaemic, compared with 66.7% (65.4-68.0) among those without diarrhoea, an absolute gap of more than 6 percentage points.

## Discussion

In this nationally representative sample of 179,262 children aged 6-59 months from India, approximately two‑thirds were anaemic. It confirms that childhood anaemia remains a severe public health problem in India despite ongoing programmes. Recent studies also reported child anaemia prevalence of 60-70% [23-27]. Bivariate results showed a higher proportion of anaemia among children with ARI, fever, and especially diarrhoea than among those without these morbidities. The positive association between diarrhoeal illness and childhood anaemia observed in this study also aligns with earlier work from India and other LMICs [14,23,27-30].

The findings from multivariable analyses show that childhood anaemia tends to cluster with recent episodes of common childhood morbidities, particularly diarrhoea. Even after adjustment for bio-demographic and socio-economic variables, childhood diarrhoea remained a significant predictor of anaemia among children (OR about 1.09 in the fully adjusted model). It indicates a statistically significant but modest association between childhood diarrhoea and anaemia. Besides, fever showed a smaller but significant association with childhood anaemia. On the other hand, the association between ARI and childhood anaemia was largely explained by confounding. The clustering of anaemia is associated with diarrhoea and, to a lesser extent, fever observed in this study reflects significant determinants of anaemia among children [27,29,30]. Moreover, predicted probabilities derived from the models reinforce these patterns, with the probability of anaemia rising from 66.7% among children without diarrhoea to 73.0% among those with diarrhoea, and more modest differences for fever and ARI.

The results also highlight early childhood, low maternal education, socio-economic disadvantage, and residence in specific high-burden regions as key variables along which the risk of anaemia is concentrated. The analysis confirmed strong age gradients; compared with children aged 6-23 months, those aged 24-41 and 42-59 months had substantially lower odds of anaemia. It indicates that the late infancy and early toddler period are the times of maximum vulnerability. This is consistent with global and Indian evidence that anaemia peaks in the second year of life, coinciding with rapid growth and frequent childhood infections [3,8,18,23,24]. The odds of childhood anaemia increased stepwise with decreasing maternal education. It corroborates earlier findings that maternal literacy shapes child diet, healthcare use, hygiene practices, and uptake of preventive services, all of which influence anaemia risk [23,24,26,29]. The strong effects of maternal age at delivery suggest that intergenerational and caregiving pathways are also important. Children born to adolescent mothers may be more likely to experience intrauterine growth restriction; low weight predisposes them to both anaemia and infection [23,28]. The association of childhood anaemia with extremes of perceived birth size (very small and very large) may capture underlying growth disturbances. It can have lasting effects on childhood anaemia [24,29].

Anaemia was more common in rural areas, among children from SC and ST households, and in poorer wealth quintile households. The higher odds among SC and ST children, even after adjustment, reflect cumulative disadvantages in living conditions, diet quality, access to services, and exposure to infections [22,23,26,29,31]. Regionally, the elevated odds of anaemia in the west, north, central, and east compared with the south are consistent with previous studies [7,25]. These studies showed persistent high-burden clusters in the Hindi-speaking belt and parts of western India and comparatively better outcomes in several southern states [3,7,23]. At the same time, the lower odds observed in the Northeast region may reflect distinct dietary patterns, lower fertility, and, in some states, relatively better maternal and child nutrition indicators [1,2,7].

Strengths and limitations

A key strength of this study is the use of large, nationally representative NFHS‑5 data, which enabled precise estimates and adjustment for a broad range of biodemographic and socioeconomic covariates. The modeling strategy progressively adds blocks of variables and estimates predicted probabilities. It helps disentangle direct and confounded associations between morbidities and anaemia and offers interpretable effect sizes for policy audiences. However, the cross-sectional design of NFHS‑5 limits causal inference, as morbidity and haemoglobin were measured at roughly the same time, and bidirectional influences cannot be fully untangled. Morbidity data are based on maternal recall over the preceding two weeks and may be affected by misclassification and reporting bias. The haemoglobin measurement captures only a single point in time and does not distinguish between iron‑deficiency anaemia and other causes. Despite these limitations, the study adds important evidence that anaemia and common childhood infections coexist in substantial proportions of Indian children and are concentrated in socioeconomically and regionally disadvantaged groups.

## Conclusions

This study demonstrates that childhood anaemia in India remains highly prevalent and is closely intertwined with common acute morbidities and deep‑rooted socioeconomic disparities. We found that about two‑thirds of children were anaemic. At the same time, a non‑trivial proportion of children experienced recent diarrhoea, fever, or ARI, and these morbidities tended to co‑occur with childhood anaemia, particularly diarrhoeal illness. The study also reveals pronounced social and regional structuring of anaemia. Anaemia burden was greatest among children aged 6-23 months, those of higher birth order, children perceived as very small at birth, and those born to adolescent or less educated mothers, pointing to the critical role of early life conditions and caregiving environments. Rural residence, children of SC and ST groups, lower wealth quintiles, and residence in the northern, central, eastern, and parts of western India were all associated with substantially higher odds of anaemia, even after extensive adjustment.

These findings argue for a shift from vertical, nutrient‑centric approaches towards integrated strategies that jointly address childhood infections and the broader social determinants of child health. In practical terms, this means prioritizing children 6-23 months from poor, rural, SC/ST, and low‑education households in high‑burden regions. The consistency and magnitude of associations observed here suggest that tackling diarrhoeal disease and social disadvantage groups alongside interventions in high-burden regions is essential. Besides, monitoring indicators that capture the overlap of anaemia with diarrhoea or ARI, rather than considering each condition in isolation, could also help track progress towards more holistic child health.

## Appendices

**Table 4:** Partially adjusted binary logistic regression estimates of the effect of childhood morbidity variables on the prevalence of anaemia in the study population by adjusting for the bio-demographic factors, NFHS-5 (2019-21) ref.: reference category

Variables		Anaemia Status								
		Model-1: Child morbidity			Model-2: Model 1 + Child level			Model-3: Model-2 + Mother level		
		OR	95% CI	p-value	OR	95% CI	p-value	OR	95% CI	p-value
Childhood morbidity variables										
Acute respiratory infection (ARI)	No (ref.)	1.00	-	-	1.00	-	-	1.00	-	-
	Yes	1.17	1.10-1.25	<0.001	1.05	0.99-1.13	0.124	1.05	0.98-1.12	0.150
Fever	No (ref.)	1.00	-	-	1.00	-	-	1.00	-	-
	Yes	1.15	1.11-1.18	<0.001	1.02	0.98-1.05	0.319	1.02	0.99-1.06	0.165
Diarrhoea	No (ref.)	1.00	-	-	1.00	-	-	1.00	-	-
	Yes	1.35	1.29-1.41	<0.001	1.14	1.10-1.20	<0.001	1.12	1.07-1.17	<0.001
Bio-Demographic Variables										
Child-level variables										
Age of the child (in months)	6-23 (ref.)	-	-	-	1.00	-	-	1.00	--	-
	24-41	-	-	-	0.59	0.57-0.60	<0.001	0.58	0.56-0.59	<0.001
	42-59	-	-	-	0.35	0.34-0.36	<0.001	0.34	0.33-0.34	<0.001
Sex of the child	Female (ref.)	-	-	-	1.00	-	-	1.00	-	-
	Male	-	-	-	1.01	0.99-1.03	0.493	1.01	0.99-1.03	0.315
Birth order	Second (ref.)	-	-	-	1.00	-	-	1.00	-	-
	First	-	-	-	0.94	0.92-0.96	<0.001	0.95	0.92-0.97	<0.001
	Third or higher	-	-	-	1.10	1.07-1.13	<0.001	1.02	1.00-1.05	0.100
Preterm birth	No (ref.)	-	-	-	1.00	-	-	1.00	-	-
	Yes	-	-	-	1.04	1.00-1.07	0.026	1.03	1.00-1.07	0.050
Size of child at birth	Average (ref.)	-	-	-	1.00	-	-	1.00	-	-
	Larger than average	-	-	-	1.04	1.01-1.07	0.024	1.04	1.01-1.08	0.016
	Smaller than average	-	-	-	1.09	1.04-1.13	<0.001	1.07	1.03-1.12	<0.001
	Very large	-	-	-	1.09	1.04-1.13	<0.001	1.09	1.05-1.14	<0.001
	Very small	-	-	-	1.21	1.12-1.30	<0.001	1.17	1.09-1.26	<0.001
Mother level variables										
Mother's age at delivery	35-49 years (ref.)	-	-	-	-	-	-	1.00	-	-
	25-34 years	-	-	-	-	-	-	1.28	1.21-1.34	<0.001
	18-24 years	-	-	-	-	-	-	1.45	1.38-1.53	<0.001
	Less than 18 years	-	-	-	-	-	-	1.50	1.38-1.64	<0.001
Mother's education	12 or more years complete (ref.)	-	-	-	-	-	-	1.00	-	-
	10-11 years complete	-	-	-	-	-	-	1.11	1.08-1.15	<0.001
	8-9 years complete	-	-	-	-	-	-	1.21	1.17-1.24	<0.001
	5-7 years complete	-	-	-	-	-	-	1.34	1.29-1.39	<0.001
	<5 years complete	-	-	-	-	-	-	1.30	1.23-1.36	<0.001
	No schooling	-	-	-	-	-	-	1.65	1.59-1.71	<0.001
Mother regularly exposed to any mass media	Yes (ref.)	-	-	-	-	-	-	1.00	-	-
	No	-	-	-	-	-	-	1.08	1.06-1.10	<0.001
